# Supplementary material for: Rasch Model of the COVID-19 Symptom Checklist—A Psychometric Validation Study
Source: Viruses. 2021 Sep 3;13(9):1762. doi: 10.3390/v13091762 (PMC8471978; doi:10.3390/v13091762)
Supplement: Supplementary file 1 [file viruses-13-01762-s001.zip › Supplemental Table S3.pdf]

Supplement Table S3. Calculation details of the transformations of raw scores into metric scales (logit scales and from zero to ten); 'Diff' refers to difference; 'COM' refers to persons with comorbidities; 'IMM' refers to persons with immunosuppressive medication.

All - no split			Item 7 - Headache - COM					Item 8 - Shortness of breath - COM					Item 5 - Pain in the limbs - IMM				
Raw score	Metric scale	Zero to ten	No COM - logit	Zero to ten	COM - logit	Zero to ten	**Diff**	No COM logit	Zero to ten	COM logit	Zero to ten	**Diff**	No IMM - logit	Zero to ten	IMM - logit	Zero to ten	**Diff**
0	-4,02	0,55	-3,95	0,71	-3,86	0,82	**-0,10**	-4,03	0,53	-4,05	0,49	**0,04**	-3,95	0,69	-4,01	0,61	**0,08**
1	-3,10	1,56	-3,03	1,71	-2,94	1,82	**-0,10**	-3,11	1,55	-3,14	1,51	**0,05**	-3,03	1,70	-3,11	1,60	**0,09**
2	-2,40	2,34	-2,33	2,47	-2,24	2,57	**-0,10**	-2,41	2,33	-2,45	2,27	**0,05**	-2,33	2,46	-2,43	2,35	**0,11**
3	-1,87	2,93	-1,80	3,05	-1,72	3,14	**-0,09**	-1,88	2,92	-1,92	2,86	**0,06**	-1,80	3,04	-1,91	2,91	**0,13**
4	-1,42	3,43	-1,34	3,55	-1,27	3,62	**-0,07**	-1,42	3,43	-1,48	3,35	**0,08**	-1,35	3,53	-1,47	3,39	**0,14**
5	-1,01	3,88	-0,92	4,00	-0,87	4,06	**-0,06**	-1,00	3,89	-1,08	3,80	**0,09**	-0,93	3,98	-1,07	3,83	**0,16**
6	-0,62	4,31	-0,53	4,42	-0,49	4,47	**-0,05**	-0,61	4,32	-0,70	4,22	**0,11**	-0,55	4,41	-0,69	4,24	**0,16**
7	-0,24	4,73	-0,15	4,83	-0,12	4,87	**-0,03**	-0,23	4,75	-0,34	4,63	**0,12**	-0,17	4,82	-0,31	4,66	**0,16**
8	0,13	5,15	0,22	5,24	0,25	5,27	**-0,03**	0,15	5,17	0,03	5,04	**0,13**	0,21	5,23	0,07	5,08	**0,15**
9	0,51	5,57	0,61	5,66	0,62	5,68	**-0,02**	0,54	5,60	0,41	5,46	**0,14**	0,59	5,64	0,46	5,51	**0,14**
10	0,91	6,01	1,00	6,09	1,02	6,10	**-0,01**	0,94	6,04	0,81	5,90	**0,14**	0,99	6,08	0,88	5,96	**0,12**
11	1,34	6,48	1,43	6,55	1,44	6,56	**-0,01**	1,37	6,52	1,24	6,39	**0,13**	1,41	6,54	1,32	6,45	**0,09**
12	1,82	7,02	1,92	7,08	1,92	7,09	**-0,01**	1,85	7,06	1,74	6,94	**0,12**	1,90	7,07	1,83	7,00	**0,07**
13	2,42	7,68	2,52	7,73	2,52	7,73	**-0,01**	2,45	7,71	2,35	7,62	**0,10**	2,50	7,72	2,44	7,67	**0,05**
14	3,28	8,63	3,38	8,66	3,38	8,66	**0,00**	3,30	8,66	3,23	8,60	**0,06**	3,35	8,66	3,32	8,63	**0,03**
15	4,52	10,00	4,61	10,00	4,61	10,00	**0,00**	4,51	10,00	4,49	10,00	**0,00**	4,59	10,00	4,57	10,00	**0,00**

Item 7 - Headache - Gender					Item 14 - Rhinites/Sinffles - Gender					Item 2 - Fatigue - Edu Status				
Female - logit	Zero to ten	Male - logit	Zero to ten	**Diff**	Female - logit	Zero to ten	Male - logit	Zero to ten	**Diff**	Below - logit	Zero to ten	Above - logit	Zero to ten	**Diff**
-3,98	0,75	-3,86	0,87	**-0,12**	-3,97	0,72	-4,01	0,68	**0,04**	-3,72	0,99	-3,95	0,74	**0,25**
-3,05	1,74	-2,94	1,86	**-0,12**	-3,05	1,71	-3,10	1,66	**0,06**	-2,84	1,94	-3,02	1,74	**0,19**
-2,35	2,49	-2,24	2,60	**-0,11**	-2,34	2,47	-2,40	2,41	**0,07**	-2,17	2,66	-2,31	2,51	**0,15**
-1,81	3,07	-1,72	3,16	**-0,10**	-1,81	3,05	-1,88	2,97	**0,08**	-1,67	3,20	-1,77	3,09	**0,11**
-1,35	3,56	-1,27	3,64	**-0,08**	-1,35	3,54	-1,43	3,46	**0,08**	-1,24	3,67	-1,31	3,58	**0,08**
-0,93	4,00	-0,87	4,07	**-0,07**	-0,94	3,99	-1,02	3,90	**0,09**	-0,84	4,09	-0,90	4,03	**0,06**
-0,54	4,42	-0,49	4,48	**-0,05**	-0,55	4,41	-0,62	4,33	**0,08**	-0,47	4,50	-0,51	4,45	**0,05**
-0,16	4,83	-0,12	4,87	**-0,04**	-0,17	4,82	-0,24	4,74	**0,08**	-0,10	4,89	-0,13	4,86	**0,03**
0,22	5,24	0,25	5,27	**-0,03**	0,20	5,22	0,14	5,16	**0,06**	0,27	5,29	0,25	5,26	**0,03**
0,61	5,65	0,63	5,67	**-0,02**	0,58	5,63	0,53	5,58	**0,05**	0,64	5,70	0,63	5,68	**0,02**
1,01	6,07	1,02	6,09	**-0,02**	0,98	6,06	0,94	6,01	**0,04**	1,04	6,12	1,02	6,11	**0,01**
1,44	6,53	1,45	6,55	**-0,01**	1,41	6,51	1,37	6,48	**0,03**	1,46	6,58	1,45	6,57	**0,01**
1,92	7,06	1,93	7,06	**-0,01**	1,89	7,04	1,87	7,01	**0,02**	1,94	7,10	1,94	7,09	**0,01**
2,53	7,70	2,54	7,71	**-0,01**	2,49	7,69	2,48	7,67	**0,02**	2,54	7,74	2,54	7,74	**0,00**
3,41	8,64	3,41	8,64	**0,00**	3,37	8,63	3,36	8,62	**0,01**	3,40	8,67	3,40	8,67	**0,00**
4,68	10,00	4,68	10,00	**0,00**	4,64	10,00	4,63	10,00	**0,00**	4,63	10,00	4,63	10,00	**0,00**
