# Supplementary material for: Rasch Model of the COVID-19 Symptom Checklist—A Psychometric Validation Study
Source: Viruses. 2021 Sep 3;13(9):1762. doi: 10.3390/v13091762 (PMC8471978; doi:10.3390/v13091762)
Supplement: Supplementary file 1 [file viruses-13-01762-s001.zip › Supplemental Table S4.pdf]

Supplemental Table S4. Reporting criteria for psychometric studies according to Streiner and Kottner (2014) [1]. We underlined in the table what we modified after the peer review.

**Criterion**	**Reported in/Action taken**
Title	Should include study type (development of an instrument versus a psychometric validation study); the title was modified accordingly.
Abstract	Modified; it now includes the aim of the study, a very brief background, the design of the study, the methods that were used, including the size and composition of the group (e.g. whether patients or students were used) and when the data were collected, the results and a conclusion.
Keywords	Reported after the abstract, as requested by the journal style. We replaced the keyword “fundamental principles of measurement” with “construct validity”.
Introduction	The gap of knowledge was reformulated and the aim of the study was revised.
Methods – Description of the scale	Included at the beginning of the methods section.
Methods – Description of the participants	Included in the methods section; modified according to the comments of the reviewers.
Methods – Sample size	Should be between 200 and 1000 on some occasions; reported in the methods section.
Methods - Procedures	Varies considerably from one paper to the next; needs to include timing, manner of administration of the instrument, numbers of scorers, debriefing results, clinical judgment, if relevant; modified and reported in the methods section (selection of items and timing).

**Criterion**	**Reported in/Action taken**
Methods – Ethics	More information was included in the methods section.
Results	• Results of statistical tests should be appropriately reported: reported in the results section, as well as the tables and the figure; additional detail is included in the Appendix.<br>• Validity testing should refer to construct validity: we modified the title and included the results of each tested parameter in the results section (fit to the Rasch model, differential item functioning, unidimensionality, person-item targeting and transformation to a metric interval scale).<br>• Parameters with estimates need to include confidence intervals: reported in Table 2.
Discussion	- Further research areas are suggested.<br>- Clinical implications are now included.<br>- More limitations were added.

1. Streiner, D.L. and J. Kottner, *Recommendations for reporting the results of studies of instrument and scale development and testing.* Journal of advanced nursing, 2014. **70**(9): p. 1970-1979.
