# Supplementary material for: Rasch Model of the COVID-19 Symptom Checklist—A Psychometric Validation Study
Source: Viruses. 2021 Sep 3;13(9):1762. doi: 10.3390/v13091762 (PMC8471978; doi:10.3390/v13091762)

Supplement

Supplement Table S1. Survey questions in German (original) and English.

German (original)	English
**Bitte geben Sie Ihre aktuelle Körpertemperatur an.**	**Please enter your current body temperature.**
Ich habe kein Fieber.	I have no fever.
Ich habe eine erhöhte Körpertemperatur/Fieber.	I have a raised body temperature/fever.
**Ich fühle mich müde.**	**I feel tired.**
Ja	Yes
Nein	No
Kann ich nicht sagen	Don't know
**Ich habe Husten.**	**I have a cough.**
Ja	Yes
Nein	No
Kann ich nicht sagen	Don't know
**Ich habe Gliederschmerzen.**	**I have aching limbs.**
Ja	Yes
Nein	No
Kann ich nicht sagen	Don't know
**Ich habe Halsschmerzen.**	**I have a sore throat.**
Ja	Yes
Nein	No
Kann ich nicht sagen	Don't know
**Ich habe Kopfweh.**	**I have a headache.**
Ja	Yes
Nein	No
Kann ich nicht sagen	Don't know
**Ich fühle mich kurzatmig.**	**I feel short of breath.**
Ja	Yes
Nein	No
Kann ich nicht sagen	Don't know
**Ich habe Schüttelfrost.**	**I have chills.**
Ja	Yes
Nein	No
Kann ich nicht sagen	Don't know
**Ich habe Übelkeit und/oder Erbrechen.**	**I have nausea and/or vomiting.**
Ja	Yes
Nein	No
Kann ich nicht sagen	Don't know
**Ich habe Durchfall.**	**I have diarrhoea.**
Ja	Yes
Nein	No
Kann ich nicht sagen	Don't know
**Ich habe eine verstopfte Nase.**	**I have a nasal congestion.**
Ja	Yes
Nein	No
Kann ich nicht sagen	Don't know
**Ich muss häufig niesen.**	**I have to sneeze a lot.**
Ja	Yes
Nein	No
Kann ich nicht sagen	Don't know
**Ich habe Schnupfen.**	**I have a cold.**
Ja	Yes
Nein	No
Kann ich nicht sagen	Don't know
**Nehmen Sie eine Abnahme des Geschmacks und/oder des Geruchs wahr?**	**Do you notice a decrease in taste and/or smell?**
Ja	Yes
Nein	No
Kann ich nicht sagen	Don't know

Supplement Table S2. Item fit statistics sorted in an ascending order according to item location in the data set with the questionnaires filled in by the participants for the first time. The smallest (negative) item location of fatigue implies that it was the item with the highest likelihood to be affirmed, whereas fever showed the lowest likelihood. Only one item (item 3 cough) showed a fit residual below the threshold of -2.5 and a significant F-test which represented individual item misfit. Cough also had a significant chi-squared value which can be interpreted as misfit to the latent trait. The Bonferroni corrected significance level was 0.000667. Significances are highlighted in bold letters.

Item number	Description	Location	Standard error	Fit residual	F-stat	p-value	ChiSq	p-value
2	Fatigue	-2.21	0.07	1.01	0.34	0.9613	3.40	0.6386
13	Sneezing	-1.67	0.07	5.29	3.34	**0.0005**	33.67	**0.0000**
12	Nasal congestion	-1.52	0.07	-0.96	2.58	0.0061	23.59	**0.0003**
14	Sniffles/rhinitis	-1.03	0.08	-2.93	3.27	**0.0006**	27.99	**0.0000**
7	Headache	-0.91	0.08	-0.86	1.82	0.0613	20.54	0.0010
3	Cough	-0.63	0.09	-4.58	2.72	0.0040	28.74	**0.0000**
6	Soare throat	-0.18	0.10	-1.33	0.47	0.8950	8.49	0.1312
5	Pain in limbs	0.10	0.11	-1.41	1.19	0.2990	13.10	0.0225
15	Smell and taste disorders	0.25	0.11	-1.84	1.09	0.3644	12.13	0.0330
11	Diarrhea	0.33	0.12	-0.28	0.82	0.5954	9.35	0.0961
4	Dry cough/no sputum production	0.37	0.12	-3.85	4.06	**0.0001**	26.09	**0.0001**
8	Shortness of breath	0.44	0.12	-2.85	0.71	0.7016	9.72	0.0835
10	Vomiting	1.55	0.19	-2.20	0.95	0.4849	7.97	0.1579
9	Chills	2.47	0.28	-1.47	2.75	0.0036	15.80	0.0074
1	Fever	2.64	0.30	0.38	0.58	0.8132	6.12	0.2951

Supplement Figure S1. Fatigue in each age group. 1 refers to 0-9 Years (24% affirmed fatigue), 10-19 Years (65% fatigue), 20-29 Years (64% fatigue), 30-39 Years (67% fatigue), 40-49 Years (60% fatigue), 50-59 Years (49% fatigue), 60-69 Years (34% fatigue), 70-79 Years (20% fatigue), 80-89 Years (50% fatigue), ≥90 Years (100% fatigue).

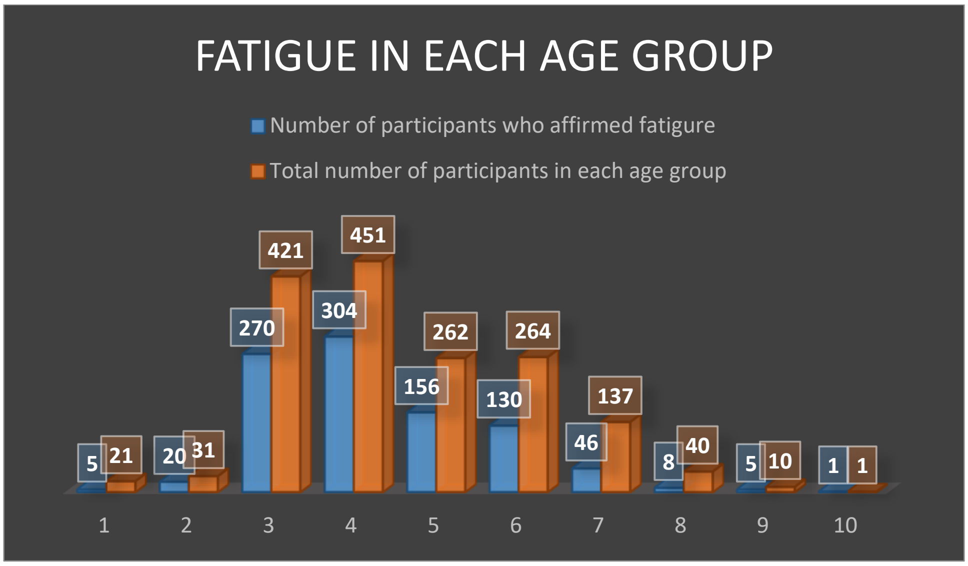

Supplement Figure S2. Person-item map. The grey bars in the top of the graph refer to the frequencies of the estimated levels of the COVID-19 symptoms of the participants (so-called person parameters). The black dot in each line represents the threshold between no symptom versus the experience of a specific symptom.

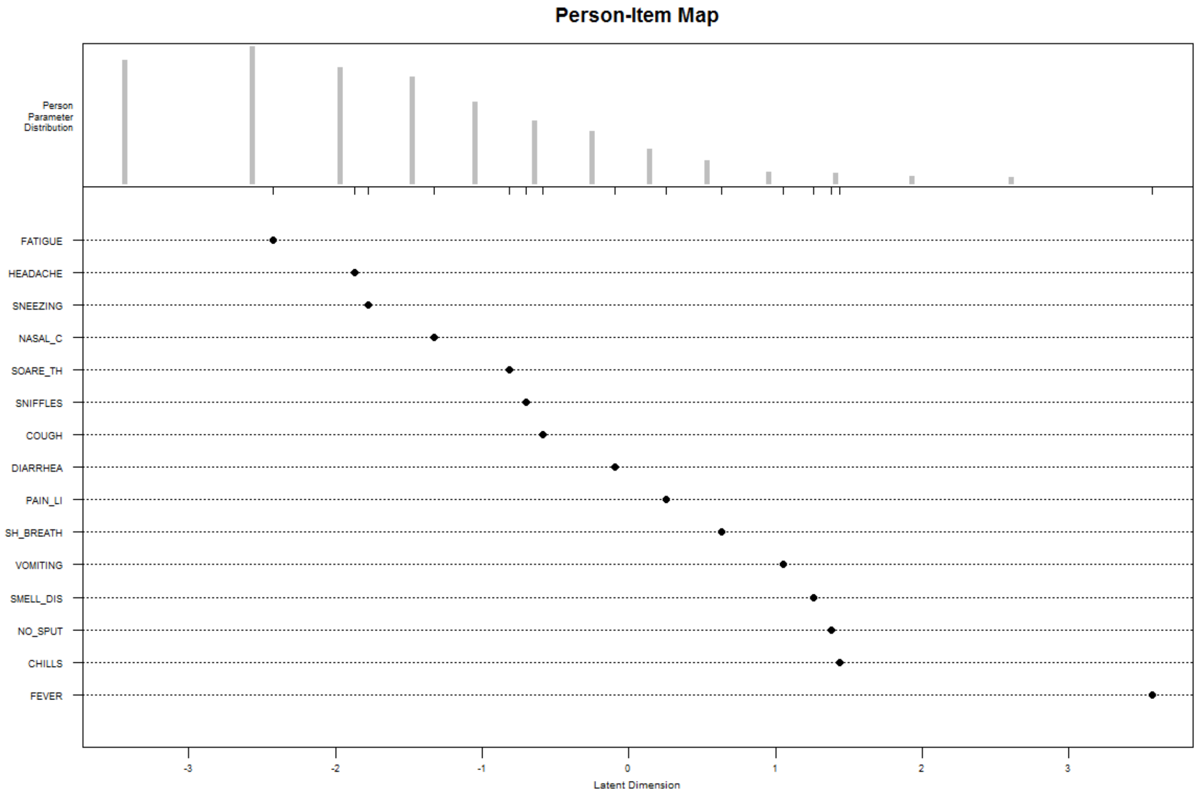

Supplement: Supplementary file 1 [file viruses-13-01762-s001.zip › Supplemental Tables S3 and S4, Figures S1 and S2.pdf]
